# Genome wide association mapping for grain shape traits in *indica* rice

**DOI:** 10.1007/s00425-016-2548-9

**Published:** 2016-05-19

**Authors:** Yue Feng, Qing Lu, Rongrong Zhai, Mengchen Zhang, Qun Xu, Yaolong Yang, Shan Wang, Xiaoping Yuan, Hanyong Yu, Yiping Wang, Xinghua Wei

**Affiliations:** 1 State Key Laboratory of Rice Biology, China National Rice Research Institute, Hangzhou, 310006 Zhejiang China; 2 Institute of Crop and Nuclear Technology Utilization, Zhejiang Academy of Agricultural Sciences, Hangzhou, 310021 Zhejiang China

**Keywords:** Genome-wide association mapping, Grain shape, Rice, Single nucleotide polymorphism

## Abstract

**Using genome-wide association mapping, 47 SNPs within 27 significant loci were identified for four grain shape traits, and 424 candidate genes were predicted from public database.**

Grain shape is a key determinant of grain yield and quality in rice (*Oryza sativa* L.). However, our knowledge of genes controlling rice grain shape remains limited. Genome-wide association mapping based on linkage disequilibrium (LD) has recently emerged as an effective approach for identifying genes or quantitative trait loci (QTL) underlying complex traits in plants. In this study, association mapping based on 5291 single nucleotide polymorphisms (SNPs) was conducted to identify significant loci associated with grain shape traits in a global collection of 469 diverse rice accessions. A total of 47 SNPs were located in 27 significant loci for four grain traits, and explained ~44.93–65.90 % of the phenotypic variation for each trait. In total, 424 candidate genes within a 200 kb extension region (±100 kb of each locus) of these loci were predicted. Of them, the cloned genes *GS3* and *qSW5* showed very strong effects on grain length and grain width in our study. Comparing with previously reported QTLs for grain shape traits, we found 11 novel loci, including 3, 3, 2 and 3 loci for grain length, grain width, grain length–width ratio and thousand grain weight, respectively. Validation of these new loci would be performed in the future studies. These results revealed that besides *GS3* and *qSW5*, multiple novel loci and mechanisms were involved in determining rice grain shape. These findings provided valuable information for understanding of the genetic control of grain shape and molecular marker assistant selection (MAS) breeding in rice.

**Electronic supplementary material:**

The online version of this article (doi:10.1007/s00425-016-2548-9) contains supplementary material, which is available to authorized users.

## Introduction

Rice (*Oryza sativa* L.) is one of the most important cereal crops and a staple food for more than one-half of the world’s population. Rice yield is directly determined by four major components: number of effective tillers per plant, number of grains per panicle, the ratio of filled grains, and grain weight (Sakamoto and Matsuoka 2008; Xing and Zhang 2010). Grain size, a key factor determining grain weight, is specified by its three dimensional structures: grain length (GL), grain width (GW), and grain thickness (GT) (Fan et al. 2006; Zuo and Li 2014). In breeding applications, grain size and weight are important traits for rice yield and quality.

Grain size and weight are generally recognized as quantitative traits that are controlled by multiple genes and affected by environment factors. To date, more than 400 QTLs that control grain size and weight have been detected by using various mapping populations (http://www.gramene.org/qtl). Several major QTLs controlling grain size and weight have been identified and functionally characterized, such as *GS3* (Fan et al. 2006; Mao et al. 2010), *GW2* (Song et al. 2007), *qSW5/GW5* (Shomura et al. 2008; Weng et al. 2008), *GS5* (Li et al. 2011), *qGL3*/*qGL3.1* (Qi et al. 2012; Zhang et al. 2012), *GW8* (Wang et al. 2012), *TGW6* (Ishimaru et al. 2013) and *GL7*/*GW7* (Wang et al. 2015a, b). Among these, *GS3* encoding a putative transmembrane protein and *qGL3*/*qGL3.1* encoding a protein phosphatase with Kelch-like repeat domain function as a negative regulator for grain length. For grain width, *GW2* encoding a RING-type E3 ubiquitin ligase and *qSW5*/*GW5* encoding a novel protein act as negative regulators, whereas *GS5* encoding a putative serine carboxypeptidase and *GW8* encoding a transcription factor with SBP domain are positive regulators. *TGW6* encodes a novel protein with indole-3-acetic acid (IAA)-glucose hydrolase activity and loss of *TGW6* function results in an increase in grain weight and yield. *GL7*/*GW7* encodes a protein homologous to *Arabidopsis thaliana* LONGIFOLIA proteins, which regulates longitudinal cell elongation. The *GL7*/*GW7* locus containing a 17.1-kb tandem duplication leads to upregulation of *GL7*/*GW7* and downregulation of its nearby negative regulator, resulting in an increase in grain length and improvement of grain appearance quality. Functional characterizations of these genes have greatly enriched our knowledge of the molecular mechanisms determining grain size and weight in rice. However, additional genes controlling grain size and weight remain to be identified.

Association mapping utilized linkage disequilibrium (LD) to examine the marker-trait associations, and enabled researchers to exploit natural variation and identify novel genes for complex traits (Zhu et al. 2008). With the development of SNP assays, high throughput genotyping technologies and associated statistical methods, association mapping has been widely used in various plant species, including *A. thaliana* (Olsen et al. 2004; Ehrenreich et al. 2009), soybean (Jun et al. 2008), wheat (Breseghello and Sorrells 2006), maize (Palaisa et al. 2003; Wilson et al. 2004; Camus-Kulandaivelu et al. 2006), foxtail millet (Jia et al. 2013) and rice (Agrama et al. 2007; Wen et al. 2009; Huang et al. 2010; Zhao et al. 2011). Association mapping has potential advantages over conventional linkage analysis and QTL mapping, such as broader allele coverage, higher mapping resolution, shorter time-consuming and improving the cost effectiveness. Association mapping has become a useful and robust strategy complementary to classical bi-parental mapping and has the power to genetically map multiple traits simultaneously (Huang and Han 2014).

In the present study, association mapping of four traits (GL, GW, grain length–width ratio (LWR) and thousand-grain weight (TGW)) was performed with a panel of 469 accessions using a custom-designed array contained 5291 genomic SNPs. The objectives of this study were to (1) investigate the population structure and genetic diversity for grain shape in a global rice germplasm collection; (2) identify the novel SNPs and loci associated with grain shape in rice; (3) explore design of gene pyramiding breeding strategies for cultivar genetic improvement. These results will increase our understanding of the molecular mechanisms underlying grain size and weight, and may provide some new information for rice molecular design breeding.

## Materials and methods

### Plant materials and phenotyping

To reduce spurious genetic associations caused by population structure, a set of 469 global diverse *indica* accessions with rice grain traits variation were chosen to construct this genome-wide association study (GWAS) population (Supplementary Table S1). The seeds of all accessions were collected, stored and supplied by China National Center for Rice Improvement, China National Rice Research Institute (CNRRI). All the rice accessions were planted in the following four environments: the Lingshui Experiment Station of CNRRI (LS; N 18°48′, E 110°02′) in Hainan, China, in the winter of 2013–2014; the Hangzhou Experiment Station of CNRRI (HZ; N 30°32′, E 120°12′) in Zhejiang, China, in the summer of 2013–2014. For field experiments, the accessions were grown in a 6 × 6 completely randomized block design with three replications. The space was 20 cm between the plants and 25 cm between the rows. Four plants in the middle of each row were harvested individually to measure the grain traits. Twenty full-filled rice grains were randomly selected from each plant for trait measurement. GL was estimated by placing 20 grains one by one in a straight line along a ruler, and then arranged by breadth to measure grain width. The averaged GL and GW of 20 grains as the trait values of that line were used for data analysis. The LWR is equal to GL divided by its GW. TGW was calculated based on 200 grains and converted to 1000-grain weight.

### DNA extraction and genotyping

Genomic DNA was extracted from leaf tissue of one plant per accession using the CTAB method (Murray and Thompson 1980). All the accessions were genotyped using the Illumina RiceSNP6k BeadChip containing 5291 SNPs, which were chosen from the Rice Haplotype Map Project Database (http://www.ncgr.ac.cn/RiceHap2) (Huang et al. 2010). Genotypes were called using the program GenomeStudio (Illumina, San Diego, Calif. USA). The quality of each SNP was confirmed manually, and low quality SNPs (call rate <80 % and minor allele frequency (MAF) <0.05) were removed from the dataset. Finally, 3951 SNPs were obtained for the association mapping (Supplementary Table S2).

### Data analysis

The mean, standard error (SE) and broad-sense heritability (*H*_*B*_^*2*^) were calculated using the Excel 2007. The percentage of phenotypic variation explained by population structure (*R*_*PCA*_^*2*^) was computed using SAS system 9.0 (SAS, Inc., Cary, NC), as well as the ANOVA. Correlation coefficients were run in R “corrgram” (https://cran.r-project.org/web/packages/corrgram/), and the best linear unbiased prediction (BLUP) was carried out in R “lme4” (https://cran.r-project.org/web/packages/lme4/) for estimating phenotypic values of each line in four environments.

### Genome-wide association mapping

For association analysis of our panel, genome association and prediction integrated tool (GAPIT) with compressed mixed linear model (cMLM) and population parameters previously defined (P3D) was performed under R environment (Lipka et al. 2012; Zhang et al. 2010). And the top 10 principle components were used as a covariant. The kinship matrix estimated by these SNPs data was combined with population structure to improve statistical power of our genome-wide association mapping. As Bonferroni correction (1/3951 = 2.5E−04) was too conservation (Nakagawa 2004), a compromised threshold of *P* = 0.001 was used to detect the significant association signals. To obtain the loci with the lowest *P* value, redundant loci were filtered in a 200 kb genomic window. And the candidate gene prediction was performed from the Rice Haplotype Map Project Database (http://202.127.18.221/RiceHap2/). Finally, the allele with an increasing effect was defined as elite allele and vice versa. The elite alleles of association loci were used to evaluate the pyramiding effect.

## Results

### Grain shape variation among accessions

**Fig. 1:**
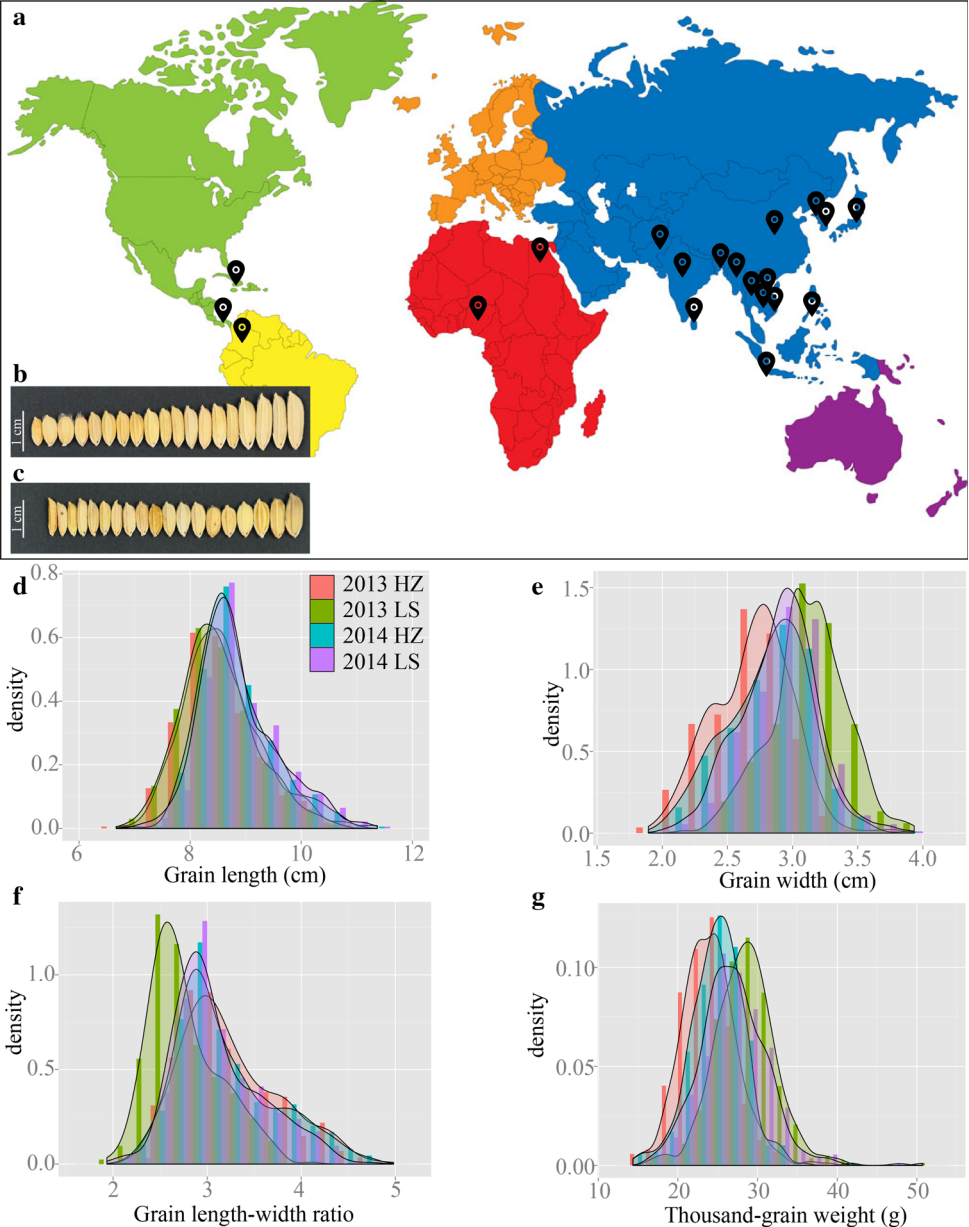
Material distribution and grain shape traits diversity. **a** The worldwide distribution of 469 *indica* accessions. The *black dots* represent the country-specific distribution. **b** Different grain length in the germplasm. *Bar* 1 cm. **c** Different grain width in the germplasm. *Bar* 1 cm. **d** Histogram of grain length in multiple environments. **e** Histogram of grain width in multiple environments. **f** Histogram of grain length–width ratio in multiple environments. **g** Histogram of thousand-grain weight in multiple environments. *LS* Lingshui, *HZ* Hangzhou

In this study, 469 *indica* accessions collected from 20 countries (Fig. 1a) were used as the genome wide association mapping panel. Four grain shape traits, namely GL, GW, LWR and TGW of 469 rice accessions were measured in four environments after harvested. Extensive and significant phenotypic variations were observed for the four grain traits in Hangzhou (HZ) and Lingshui (LS) during 2013–2014 (Table 1; Fig. 1b, c; Supplementary Figure S1; Supplementary Table S3). The mean of GL over the 469 accessions in each environment was 8.58, 8.51, 8.81 and 8.86, respectively. Over the four environments the minimum GL value was in 2013 HZ (6.67 cm), and the maximum value was in 2014 HZ (11.37 cm). GW had means of 2.69, 3.12, 2.81 and 2.89 in each environment, respectively. GW value ranged from 1.89 to 2.17 cm in the four environments. The mean of LWR over the 469 accessions in each environment was 3.24, 2.76, 3.19 and 3.10, respectively. Over the four environments the minimum LWR value was in 2013 LS (1.94), and the maximum value was in 2013 HZ (4.98). TGW had means of 23.94, 28.44, 25.04 and 26.88 g in each environment, respectively. TGW value ranged from 14.35 to 50.75 g in the four environments. The distribution of the four grain traits in all four environments showed continuous variation, and the phenotypic segregation approximately fit a normal distribution (Fig. 1d–g). This indicated that grain shape traits were governed by multiple loci in this association panel. The broad-sense heritability (*H*_*B*_^*2*^ %), averaged across four environments, of GL, GW, LWR and TGW, was 79.1, 84.4, 89.6 and 83.7 %, respectively. The ANOVA indicated that effects of genotype (G), environment (E) and their interaction were high significantly (*P* < 0.001) (Table 1).

**Table 1:** Phenotypic variation for grain traits across four environments in 469 *indica* accessions

Trait	Environment	Mean ± SE	Range	*R*_PCA_^2^ (%)^a^	*H*_B_^2^ (%)^b^	G × E^c^
GL (cm)	2013, HZ	8.58 ± 0.03	6.67–10.92	29.10	79.1	***
	2013, LS	8.51 ± 0.03	6.83–10.67	27.24		
	2014, HZ	8.81 ± 0.03	6.92–11.37	31.65		
	2014, LS	8.86 ± 0.03	7.11–11.20	32.22		
GW (cm)	2013, HZ	2.69 ± 0.01	1.89–3.79	37.50	84.4	***
	2013, LS	3.12 ± 0.01	2.17–3.93	31.10		
	2014, HZ	2.81 ± 0.01	1.92–3.90	39.37		
	2014, LS	2.89 ± 0.01	2.02–3.90	32.53		
LWR	2013, HZ	3.24 ± 0.02	2.17–4.98	41.23	89.6	***
	2013, LS	2.76 ± 0.02	1.94–4.11	38.52		
	2014, HZ	3.19 ± 0.02	2.15–4.74	43.73		
	2014, LS	3.10 ± 0.02	1.97–4.75	38.26		
TGW (g)	2013, HZ	23.94 ± 0.16	14.50–38.85	19.16	83.7	***
	2013, LS	28.44 ± 0.18	16.70–50.75	21.82		
	2014, HZ	25.04 ± 0.15	14.35–41.75	18.69		
	2014, LS	26.88 ± 0.19	16.45–47.80	23.96		

*GL* grain length, *GW* grain width, *LWR* grain length–width ratio, *TGW* thousand-grain weight, *LS* Lingshui, *HZ* Hangzhou, *SE* standard error

*** Significant at *P* = 0.001

^a^Phenotypic variation explained by population structure

^b^Heritability in the broad sense

^c^G × E, interaction of genotype and environment

**Fig. 2:**
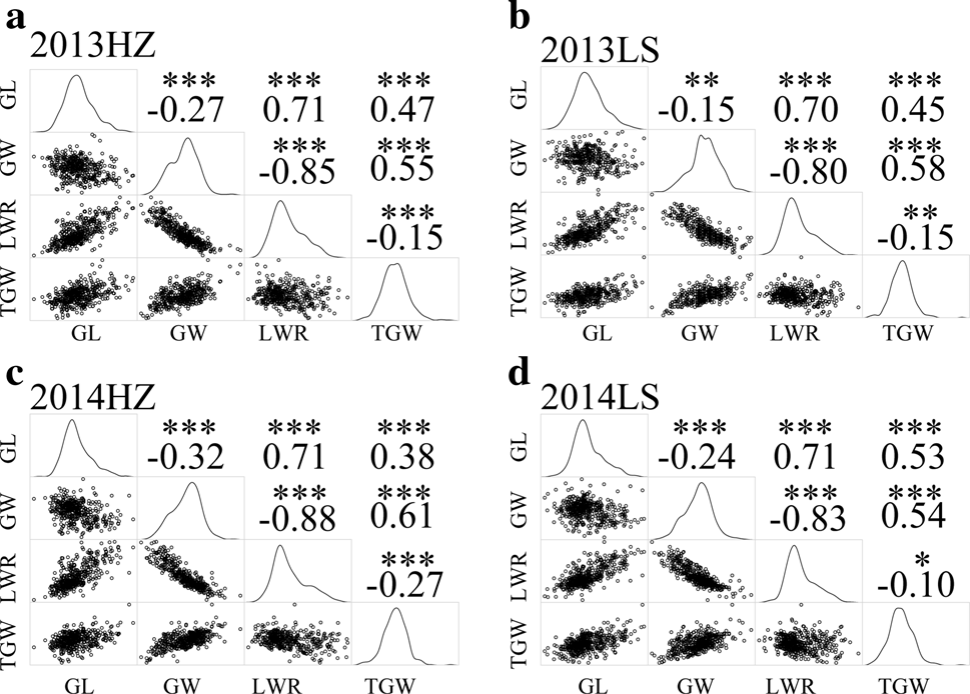
Correlation of four grain shape traits in four environments. The *upper panel* contains the correlation coefficients, and the *lower panel* contains the distributions of the four traits. The *diagonal* represents the *density line* of the traits. *, ** and *** represent significant at 0.05, 0.01 and 0.001, respectively. *GL*, *GW*, *LWR* and *TGW* represent grain length, grain width, grain length–width ratio and thousand grain weight, respectively. *HZ* and *LS* represent Hangzhou and Lingshui, respectively

The results of Pearson correlation analysis showed that the phenotypic correlations between traits were similar in the four testing environments (Fig. 2). GW showed significantly negative correlation with GL and LWR, and highly positive correlation with TGW in all environments. TGW exhibited significantly or very significantly negative correlation with LWR, and highly positive correlation with GW. The correlation coefficients ranged from -0.88 between LWR and TGW, to 0.71 between LWR and GL (Fig. 2). These results demonstrated that rice grain traits were highly related to each other, and this provided valuable knowledge for rice grain shape modifications.

### Genetic structure and linkage disequilibrium estimation

**Fig. 3:**
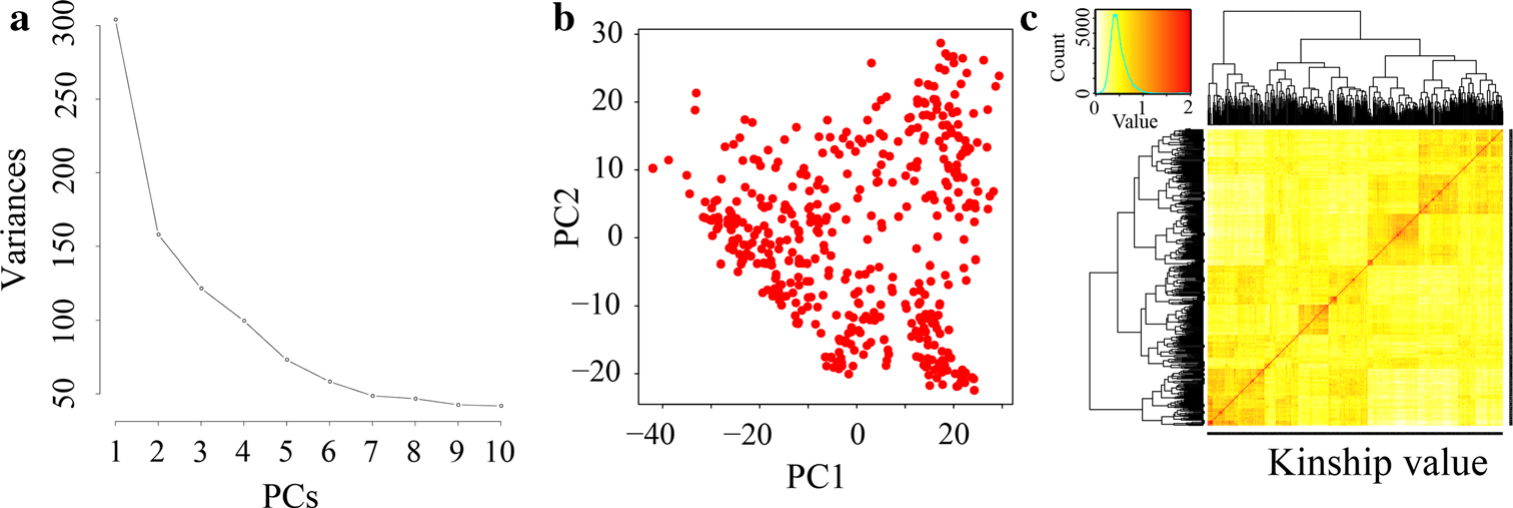
Population structure of current association *panel* which consisted mostly of the *indica* accessions. **a** Scree plot from GAPIT showing the selection of PCs for association study. **b** Variation of the top two principal components. **c** Kinship values

STRUCTURE software was used to assess the subpopulation genetic structure of the 469 *indica* rice accessions based on the distribution of the 3,951 SNPs across 12 rice chromosomes. Our previous study showed that the *indica* panel could be classified into four subpopulations (Lu et al. 2015). In this GWAS mapping, the population effect showed by a principal component analysis (PCA) component and relatedness estimated automatically from the genotypic data were evaluated using GAPIT as well as GWAS (Lipka et al. 2012; Zhang et al. 2010). The scree plot generated through GAPIT indicated the first two PCs as informative, and then decreased gradually (Fig. 3a, b). Moreover, until the 10th PC component, the variance value almost unchanged, thus the top 10 PCs were used as a covariate to adjust the GWAS results (Fig. 3a). In addition, the heatmap of kinship value showed that most of the value concentrated at ~0.5 level indicating a weak relatedness in the complex GWAS panel (Fig. 3c). This evaluation is consistent with our previous study suggesting that the GAPIT is credible for population structure and relative kinship estimations (Lu et al. 2015). In the present study, the linkage disequilibrium (LD) decay distance was ~109.37 kb at which the LD parameter (*r*^2^) dropped to half of its maximum value. In addition, the LD decay distance differed among chromosomes and ranged from 96.15 kb on chromosome 5 to 421.39 kb on chromosome 7.

### Genome-wide association analysis

**Fig. 4:**
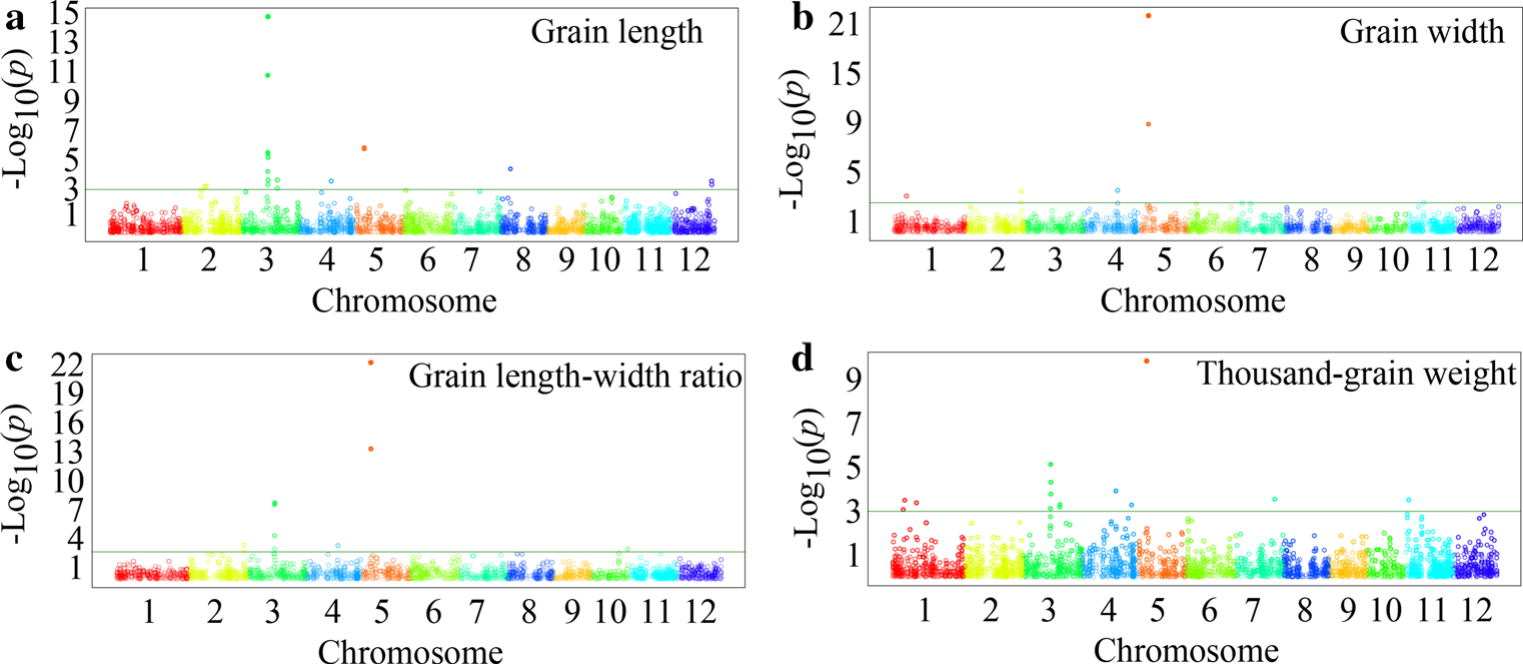
Manhattan plots of genome wide association mapping for four rice grain traits. **a** Grain length, **b** grain width, **c** grain length–width ratio, **d** thousand grain weight. *Green lines* represent the threshold at −log_10_ (*P*) ≥3

In this study, the association analysis was performed using BLUP method predicted for each accession to reduce the environment effects and simplify the calculations. The stringent criterion of −log_10_ (*P*) ≥3.0 under four environments was used for determining the association significance of the four grain traits. Using the GWAS approach, we successfully identified both known genes and previously reported QTLs from rice as well as novel candidate loci in the rice genome. The results of our genome-wide association scans were summarized in Fig. 4 and Table 2 where we showed the SNP trait associations discovered in the association panel. A total of 27 significant loci were identified for the four grain traits. For the 27 significant loci, 424 candidate genes were obtained within a 200-kb genomic region (±100 kb from the peak SNP) from the Rice Haplotype Map Project Database (http://202.127.18.221/RiceHap2/) (Supplementary Table S4). In addition, the distributions of the 3951 SNPs were analyzed and 47 SNPs were located in the 27 significant loci for the four grain traits (Supplementary Table S2, Table S4). Of the 27 loci, *GS3* and *qSW5* showed very strong effects on grain length and width. Simultaneously, in order to investigate environment-specific and common loci under multiple environments, we performed association mapping using TASSEL version 4.0 (Bradbury et al. 2007) under each environment (Supplementary Table S5).

**Table 2:** Summary of SNPs significantly associated with grain traits using the BLUP method

Trait	Marker	Chr.	Position	Allele^a^	MAF	*P* value	*R* ^2^ (%)^b^	Known QTL	Marker	References
GL	seq-rs918	2	13760905	G/A	0.17	6.68E−04	50.23			
	seq-rs919	2	14722011	T/G	0.17	6.68E−04				
	seq-rs1614	3	16939138	T/C	0.25	1.87E−15		*GS3*		Fan et al. (2006)
	seq-rs1697^c^	3	22579680	G/T	0.21	2.63E−04		*qGL3a*	RM15456	Li et al. (2010)
	seq-rs2123	4	19733128	C/T	0.05	2.89E−04		*qGL4b*	RM5586	Kato et al. (2011)
	gwseq-rs14^d^	5	5377176	T/C	0.21	1.91E−06		*qSW5*		Shomura et al. (2008)
	seq-rs3750	8	5964991	C/T	0.06	4.35E−05				
	seq-rs5920	12	25594275	G/A	0.36	2.91E−04		*qGL12*	RM2854	Li et al. (2010)
GW	seq-rs196	1	7562923	A/G	0.29	2.24E−04	58.57			
	seq-rs1303	2	33850349	T/C	0.25	7.62E−05				
	seq-rs2139	4	21062959	A/G	0.42	6.02E−05				
	seq-rs2427^d^	5	5359498	G/A	0.32	1.12E−22		*qSW5*		Shomura et al. (2008)
LWR	seq-rs1303	2	33850349	T/C	0.25	2.75E−04	65.90			
	seq-rs1614	3	16939138	T/C	0.25	9.46E−09		*GS3*		Fan et al. (2006)
	seq-rs2123	4	19733128	C/T	0.05	3.03E−04		*qLWR4*	RM6997	Ying et al. (2012)
	seq-rs2427	5	5359498	G/A	0.32	1.48E−23		*qSW5*		Shomura et al. (2008)
	seq-rs4717	10	22631657	T/C	0.08	7.37E−04				
TGW	seq-rs184	1	6667379	A/G	0.45	9.17E−04	44.93	*Gw1*-*1*	RM10398	Yu et al. (2008)
	seq-rs196	1	7562923	A/G	0.29	3.70E−04		*QGwt1c*, *qBRW1a*	RM259	Xu et al. (2002), Wang et al. (2003)
	seq-rs288	1	14827946	C/T	0.30	4.76E−04				
	seq-rs1614	3	16939138	T/C	0.25	9.00E−06		*GS3*		Fan et al. (2006)
	seq-rs1698^c^	3	22582110	C/A	0.21	5.42E−04		*qTGW3*-*4*, *qGW3.8*	RM16	Marathi et al. (2012), Zhang et al. (2013)
	seq-rs2139	4	21062959	A/G	0.42	1.41E−04				
	seq-rs2255	4	30599877	A/G	0.18	5.67E−04		*qTGW4*-*1*	RM3276	Marathi et al. (2012)
	seq-rs2427	5	5359498	G/A	0.32	2.03E−10		*qSW5*		Shomura et al. (2008)
	seq-rs3526	7	23662096	C/A	0.07	3.19E−04				
	seq-rs4745	11	3031650	T/C	0.34	3.40E−04		*GWt11*	RM332	Yoshida et al. (2002)

*GL* grain length, *GW* grain width, *LWR* grain length–width ratio, *TGW* thousand-grain weight

^a^Major allele/minor allele

^b^Phenotypic variation explained by all of the significant loci

^c^The two SNPs can be considered as the same locus (~2 kb)

^d^The two SNPs can be considered as the same locus (~17 kb)

**Fig. 5:**
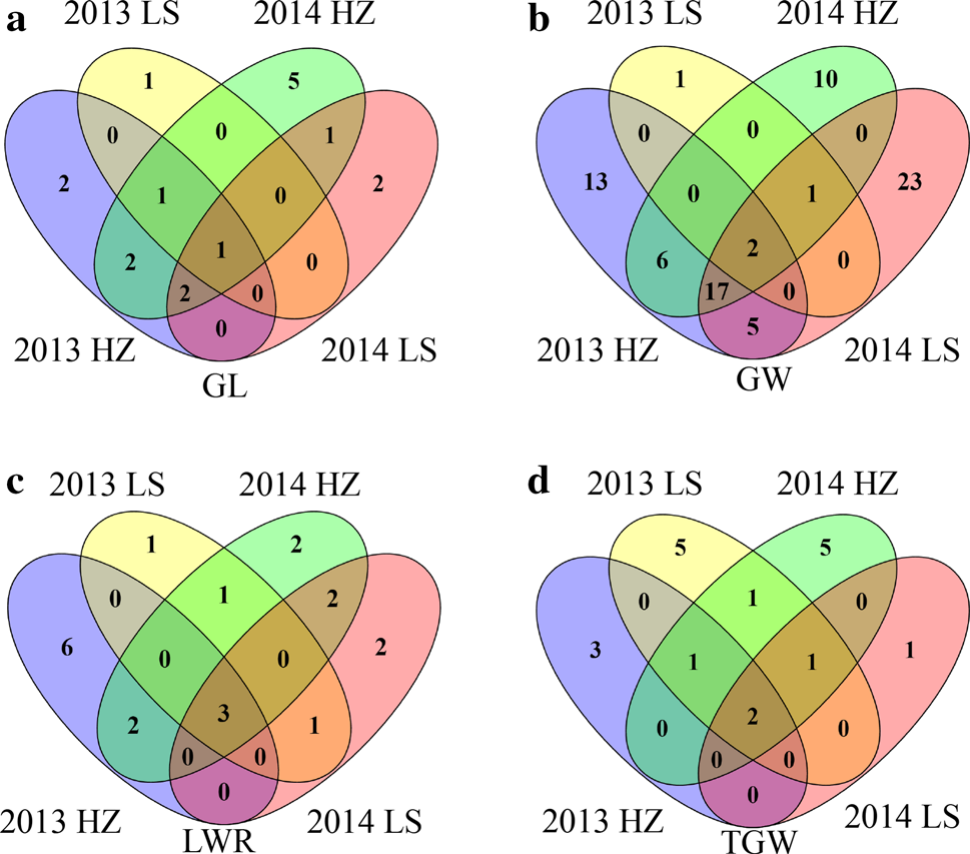
Venn diagrams showing unique and shared SNPs for grain shape traits among four environments. **a** *GL* grain length. **b** *GW* grain width. **c** *LWR* grain length–width ratio. **d** *TGW* thousand grain weight. *LS* and *HZ* represent Lingshui and Hangzhou, respectively

For the GL trait, 8 major loci, explaining ~50.23 % of the phenotypic variation, were identified on chromosomes 2, 3, 4, 5, 8 and 12 (Table 2). Among them, the *GS3* locus (seq-rs1614) was confirmed and explained ~8.50–14.73 % of the total phenotypic variation under four environments (Fig. 5a; Supplementary Table S6), and this result was consistent with previous reports (Huang et al. 2010; Zhao et al. 2011). Furthermore, the loci, seq-rs1610, seq-rs2061 and seq-rs4660, on chromosomes 3, 4 and 10 were identified across three environments (Fig. 5a; Supplementary Table S6). Additionally, the loci, seq-rs1604 and seq-rs2427, on chromosomes 3 and 5 were detected in both 2013 and 2014 HZ environments (Fig. 5a; Supplementary Table S6). The locus (seq-rs2718) on chromosome 6 was identified in both 2014 HZ and LS environments (Fig. 5a; Supplementary Table S6).

For the GW trait, 4 major loci, explaining ~58.57 % of the phenotypic variation, were identified on chromosomes 1, 2, 4 and 5 (Table 2). Among them, the previously cloned *qSW5* locus showed very strong effect on GW and had high *R*^2^ values (~13.6–27.79 %) in the four environments (Supplementary Table S5), and this result was consistent with previous reports (Huang et al. 2010; Zhao et al. 2011). The locus (seq-rs2434) as well as the *qSW5* (seq-rs2427), on chromosome 5 was detected across four environments (Fig. 5b; Supplementary Table S6). In addition, eleven and eighteen significant loci were identified across two and three environments, respectively (Fig. 5b; Supplementary Table S6). Moreover, the loci, seq-rs196, seq-rs1303 and seq-rs2139, on chromosomes 1, 2 and 4 were also detected for TGW, LWR and TGW, respectively (Table 2).

In our study, marker-trait association analyses revealed that 5 loci associated with LWR, locating on chromosomes 2, 3, 4, 5 and 10 (Table 2; Fig. 4c). These loci could explain up to 65.90 % of the total phenotypic variation (Table 2). Among them, the known genes *GS3* and *qSW5* were showed clear signal for LWR in all the four environments (Fig. 5c; Supplementary Table S6). The locus (seq-rs1610) on chromosome 3 was also detected across four environments (Supplementary Table S6). Additionally, the loci (seq-rs944 and seq-rs2123) on chromosomes 2 and 4 were detected in both 2013 and 2014 HZ, and the loci (seq-rs3533 and seq-rs3537) on chromosome 7 were identified in both 2013 and 2014 LS (Fig. 5c; Supplementary Table S6).

For the TGW trait, 10 major loci, explaining ~44.93 % of phenotypic variation, were detected on chromosomes 1, 3, 4, 5, 7 and 11, and six loci were located in the adjacent or overlapping regions previously reported to be associated with TGW (Table 2). Moreover, the loci (seq-rs288 and seq-rs2139) on chromosomes 1 and 4 were identified across three environments (Fig. 5d; Supplementary Table S6). Furthermore, we also identified the known genes (*GS3* and *qSW5*) for TGW in all four environments (Fig. 5d; Supplementary Table S6). Additionally, the locus (seq-rs5793) on chromosome 12 was detected in both 2013 LS and 2014 HZ (Fig. 5d; Supplementary Table S6).

### Gene linkage or pleiotropy

**Fig. 6:**
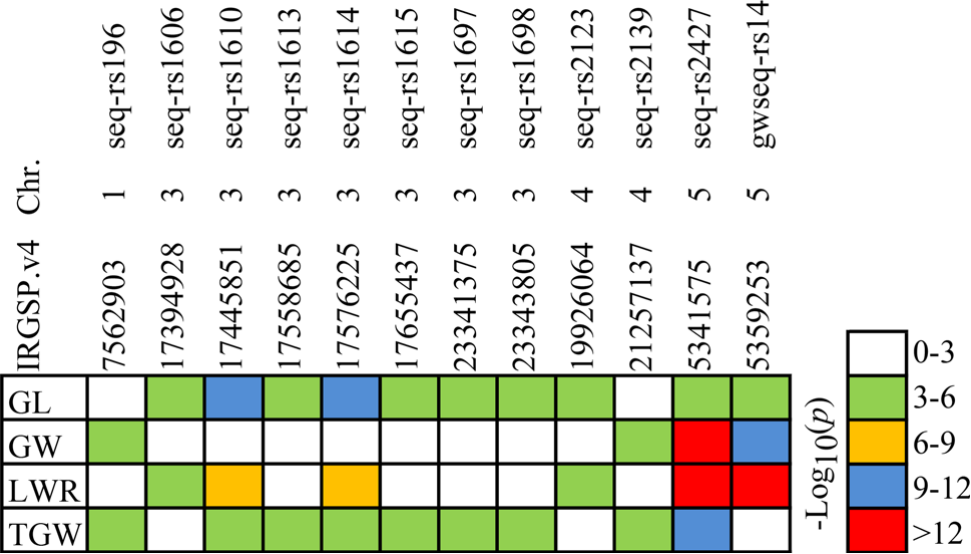
Summary of significant trait-marker associations for four grain traits across genomic regions. *GL*, *GW*, *LWR* and *TGW* represent grain length, grain width, grain length–width ratio and thousand grain weight, respectively

Gene linkage and pleiotropy are common phenomena in plant genetics. A matrix summarizing the linkage or pleiotropy of the loci associated with four grain traits were shown in Fig. 6. In our study, 12 known or new candidate SNPs showed clear signal linkage or pleiotropy that was associated with multiple grain traits in the four environments. Among them, the loci seq-rs1614 and seq-rs2427 underlying grain size (*GS3* and *qSW5*) were shown to be significantly associated with GL, LWR, TGW and all four grain traits, respectively (Fig. 6). Previous studies showed that *GS3* had pleiotropic effects on grain length and weight (Fan et al. 2006; Mao et al. 2010), and *qSW5* was associated with grain width and weight (Shomura et al. 2008; Weng et al. 2008). These associations were also supported by Pearson correlation analysis based on traits measured in all four environments (Fig. 2). In addition, we detected the locus (seq-rs2123) on chromosome 4 that were significantly associated with both GL and LWR, and we found a locus (seq-rs196) on chromosome 2 that was significantly associated with GW and TGW. Moreover, the average correlation coefficients of GL-LWR (*r* = 0.71, *P* < 0.001) and GW-TGW (*r* = 0.57, *P* < 0.001) were highly significant in all four environments. Additionally, some new candidate loci possessing gene linkage or pleiotropy were observed as well (Fig. 6). Exploring and utilizing the linkage or pleiotropy of the loci underlying grain traits would be beneficial for rice grain yield improvement.

### Pyramiding elite alleles for breeding

**Fig. 7:**
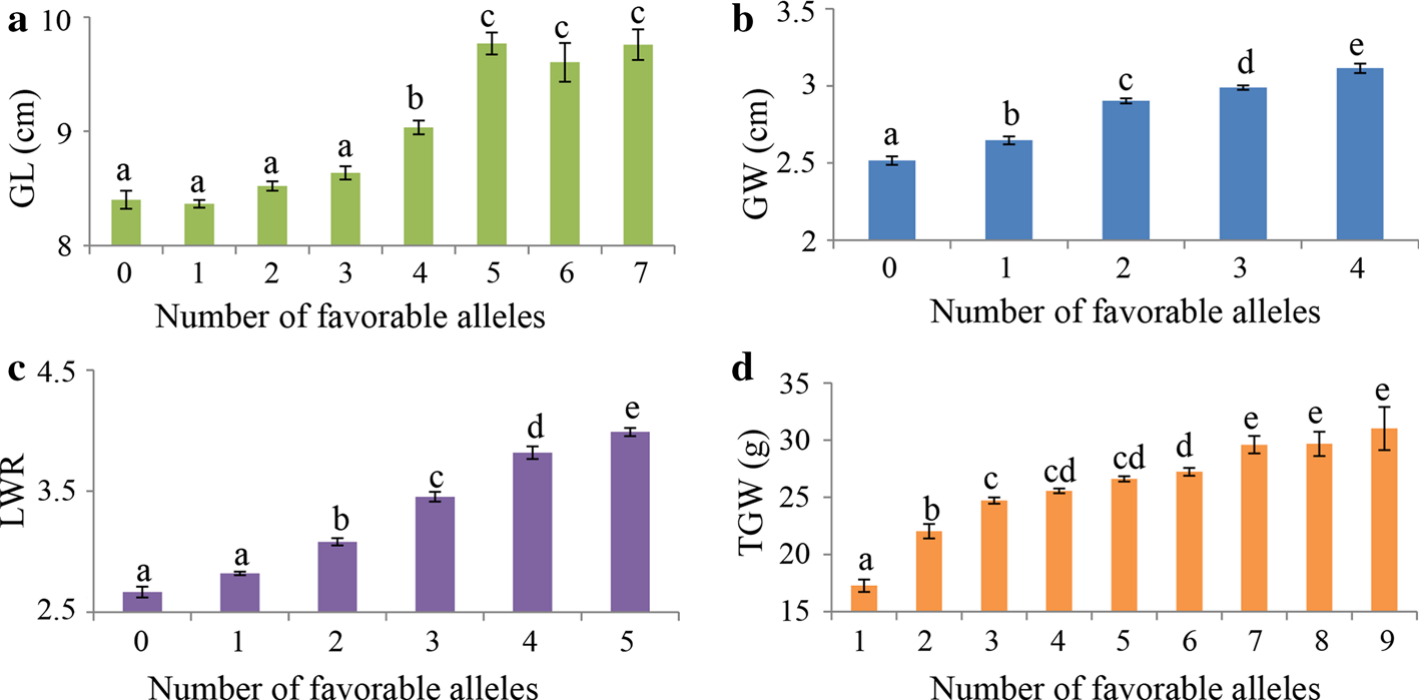
Pyramid effect analysis for different numbers of elite alleles. *X-axis* represents the number of elite alleles carried by the accessions and *Y-axis* represents trait mean value *GL*, *GW*, *LWR* and *TGW* represent grain length, grain width, grain length–width ratio and thousand grain weight, respectively. All *bars* represent standard error

The objective of gene pyramiding in molecular breeding is to combine a series of target alleles in a specific line or variety (Servin et al. 2004). We examined the efficacy of pyramiding elite alleles into an individual plant for four grain traits (Supplementary Table S7). Besides *GS3* (seq-rs1614) and *qSW5* (seq-rs2427), such as the eilte allele of the locus seq-rs1698 showed potential breeding value for GL and TGW (Supplementary Table S7). This result was also confirmed by classical QTL mapping in different bi-parental mapping populations (Li et al. 2010; Marathi et al. 2012; Zhang et al. 2013). Without considering the interaction effects among these loci, mean values for phenotypic traits except GL significantly increased linearly with pyramiding more elite alleles in rice cultivars (Fig. 7). For the GL trait, when pyramiding 0–3 elite alleles, the mean phenotypic values had no significant changes, and the mean phenotypic values increased significantly with pyramiding 4 elite alleles, and the mean phenotypic values were significantly higher when pyramiding 5–7 elite alleles in rice varieties (Fig. 7). These results indicated that enhancing the frequency of elite alleles has a significant phenotypic effect on rice grain traits improvements.

## Discussion

Rice is one of the most staple foods in the world, feeding more than half of the global population especially in Asian countries. It has been estimated that a 40 % increase in rice production by 2030 will be needed to meet the predicted demand of the growing world population (Khush 2005). Rice grain shape is one of the most important factors determining rice yield (Huang et al. 2013). Thus, identification of major QTLs for grain shape and grain weight is an important objective of rice genetic research and breeding programs. In this study, genome-wide association mapping was performed to identify the loci for four grain shape traits in 469 rice accessions. The sample size of our study is larger or similar to the number of accessions used in genome wide association study in rice (Huang et al. 2010; Zhao et al. 2011). The 469 diverse accessions were from 20 countries, which represented all major rice growing regions of the world (Fig. 1a). Four grain shape traits including GL, GW, LWR and TGW were measured in four environments. We successfully identified 27 loci for the four grain traits, comprising 17 distinct regions distributed on all 12 chromosomes except chromosomes 6 and 9.

In rice, more than 400 QTLs that control grain shape traits have been detected by using various mapping population (Huang et al. 2013). To date, 20 genes associated with grain size and weight have been isolated by map-based cloning strategies (Huang et al. 2013; Zuo and Li 2014; Duan et al. 2013; Hu et al. 2015; Liu et al. 2015a, b; Wang et al. 2015a, b). We compared the positions of the significant loci identified in this study with the positions of the reported genes or QTLs for the four grain traits. Of the 27 identified loci, the positions of 16 loci were close to those reported in previous studies, including 5, 1, 3 and 7 loci for GL, GW, LWR and TGW, respectively (Table 2).

As is known, *GS3* and *qSW5* are major QTLs for GL and GW (Fan et al. 2006; Shomura et al. 2008), which showed very strong effect for the same phenotypes in our study. This result was consistent with the GWAS results for GL and GW by Huang et al. (2010) and Zhao et al. (2011). Kato et al. (2011) mapped a GL QTL (*qGL4b*) to a 3.0 Mb region on chromosome 4 between marker RM5586 and RM3524. Interestingly, we detected a locus (seq-rs2123) for both GL and LWR in this chromosome region (Table 2), within which overlapped with a QTL for LWR reported by Ying et al. (2012). In addition, we detected 2 loci (seq-rs1697 and seq-rs5920) for GL on chromosome 3 and 12, respectively (Table 2), which have also been located in similar positions in different mapping population (Li et al. 2010). Moreover, Yu et al. (2008) mapped a TGW QTL (*Gw1*-*1*) to a 392.9 kb region on chromosome 1 between marker RM10376 and RM10398. We also detected a locus (seq-rs184) for TGW in the overlapped region. In the present study, 2 significant loci (seq-rs1698 and seq-rs2255) for TGW were located in the same genomic regions as those previously reported by Marathi et al. (2012). Additionally, three out of eight associated loci for TGW were mapped in adjacent intervals on the same chromosome as those reported in previous studies (Table 2). These results demonstrated that our 6K SNP array could capture the major common variants responsible for grain shape traits. However, no significant loci for grain traits were detected on chromosomes 6 and 9. One possible explanation was that the QTL effects were too small to be detected in our association panel, and another reason might be due to the lack of SNP coverage in the potential QTL regions. On the other hand, we identified 11 novel loci, including 3, 3, 2 and 3 loci for GL, GW, LWR and TGW, respectively (Table 2). Validation of these new loci would be performed in the future studies.

QTL× environment interaction is an important component for quantitative traits (Xing et al. 2002). In the present study, we observed some significant loci were only detected in one environment. For example, the associated loci, seq-rs3156, seq-rs4859, seq-rs768 and seq-rs2255 for GL, GW, LWR and TGW, respectively, were only detected in 2013 HZ. Result of ANOVA also showed that the G × E interactions were highly significant for all rice grain traits. Therefore, the QTL× environment interaction should not be ignored if molecular marker assistant selection (MAS) is applied for the manipulation of rice grain shape traits. However, the stable expression of a QTL across a broad range of agrometeorological conditions is a critical factor when breeding for wide adaptation (Maccaferri et al. 2008). In this study, the major loci (*GS3* and *qSW5*) were detected across all four environments. These results were also proved by those obtained NILs carrying various alleles in the same background of ZhenShan97 (Lu et al. 2013) and supported by other GWAS experiments (Huang et al. 2010; Zhao et al. 2011). These results suggested that the major loci were first fixed in cultivated variety free of environmental influences due to strong human selection. Additionally, 3, 18 and 2 significant loci for GL, GW and TGW, respectively, were detected across three environments (Fig. 5; Supplementary Table S6). Moreover, 3, 11, 6 and 1 significant loci for GL, GW, LWR and TGW, respectively, were detected across two environments (Fig. 5; Supplementary Table S6). These stable significant loci affecting grain traits across different environments may be helpful in MAS of rice grain shape breeding.

The colocation of QTLs for multiple traits may be due to either pleiotropy or tightly linked genes controlling related traits (Brown 2002; Chen and Lubberstedt 2010). Our results showed that the associated loci *GS3* and *qSW5* exhibited strong pleiotropic effects on grain size and grain weight. Besides, 5 overlapped regions (seq-rs2123, seq-rs184, seq-rs196, seq-rs1698 and seq-rs2255) were found to be associated with grain traits in this study (Table 2). Among them, the locus (seq-rs2123) was detected for both GL and LWR, which was closely to the position identified by Kato et al. (2011) for GL and Ying et al. (2012) for LWR. In addition, the seq-rs1697 locus was detected for GL and TGW on chromosome 3. Its position was coincident with previously identified QTLs (Li et al. 2010; Marathi et al. 2012). Moreover, we identified a new locus (seq-rs2139) on chromosome 4 for GW and TGW across three environments. The Pearson correlation analysis also proved that the traits with co-localized loci were correlated with each other in all four environments (Fig. 2). Accumulation of favorable alleles in linkage blocks is very important and useful for breeders to implement grain shape improvement programs. Characterization and validation studies involving joint linkage and association mapping, combining with fine mapping to identify the novel genes and alleles underlying our association hits, would help us more clearly understand the relationship between these candidate genes and the phenotypes observed in our study, and provide breeders with the effective genetic tools to break unfavorable linkages and exploit these valuable alleles.

In the present study, we identified 27 loci associated with four grain traits via GWAS and convincingly demonstrated that the rice grain shape is complex trait controlled by many genes of major or minor effect. We also refined candidate chromosomal regions for the known QTLs, including the cloned genes *GS3* and *qSW5*. Moreover, new candidate loci were obtained, and genetic variations combined with phenotypic variances were observed. Results of the present study demonstrated that genome-wide association mapping in rice could complement and enhance the information from linkage-based QTL studies toward the implementation of MAS in breeding programs. Considering the effect and distribution of novel loci associated with grain shape in our study, further validation is needed to unearth the relationship between these candidate loci and the phenotypes and facilitate exploring the molecular mechanisms governing grain shape suitable for rice breeding programs.

### *Author contribution statement*

YF and XHW designed the experiments. YF, QL, RRZ and MCZ performed the phenotyping. QX, YF, RRZ and YLY carried out the genetic studies. XPY, HYY and YPW managed the materials. XHW designed the overall project. YF analyzed the data and drafted the manuscript. SW and YLY helped to revise the manuscript. All of the authors read and approved the final manuscript.

## Electronic supplementary material

Below is the link to the electronic supplementary material.

Supplementary Figure S1: Box plot of four grain shape traits in four environments. (a) GL, grain length. (b) GW, grain width. (c) LWR, grain length–width ratio. (d) TGW, thousand grain weight. LS and HZ represent Lingshui and Hangzhou, respectively (TIFF 471 kb)

Supplementary Table S1: The origin of 469 *indica* accessions (XLSX 25 kb)

Supplementary Table S2: Summary of 3951 SNPs information (XLSX 147 kb)

Supplementary Table S3: Phenotypic data for four grain traits in Hangzhou and Lingshui during 2013–2014 (XLSX 107 kb)

Supplementary Table S4: Summary of 424 candidate genes for four grain traits (XLSX 38 kb)

Supplementary Table S5: Summary of SNPs significant associated with four grain traits in Hangzhou and Lingshui during 2013–2014 (XLSX 24 kb)

Supplementary Table S6: Summary of common loci associated with grain shape traits under multiple environments (XLSX 16 kb)

Supplementary Table S7: Summary of elite allele and phenotypic effect (XLSX 16 kb)

## Abbreviations

GL: Grain length
GW: Grain width
GT: Grain thickness
TGW: Thousand grain weight
LWR: Grain length–width ratio
QTL: Quantitative trait loci
LD: Linkage disequilibrium
SNP: Single nucleotide polymorphism
GWAS: Genome-wide association study
CTAB: Hexadecyltrimethy ammonium bromide
MAF: Minor allele frequency
SE: Standard error
ANOVA: Analysis of variance
BLUP: Best linear unbiased prediction
cMLM: Compressed mixed linear model
PCA: Principle component analysis
G × E: Interaction of genotype and environment
GAPIT: Genome association and prediction integrated tool
MAS: Molecular assistant selection
