# Supplementary figures and images for: Genome wide association mapping for grain shape traits in indica rice
Source: Planta. 2016 May 19;244(4):819–30. doi: 10.1007/s00425-016-2548-9 (PMC5018019; doi:10.1007/s00425-016-2548-9)

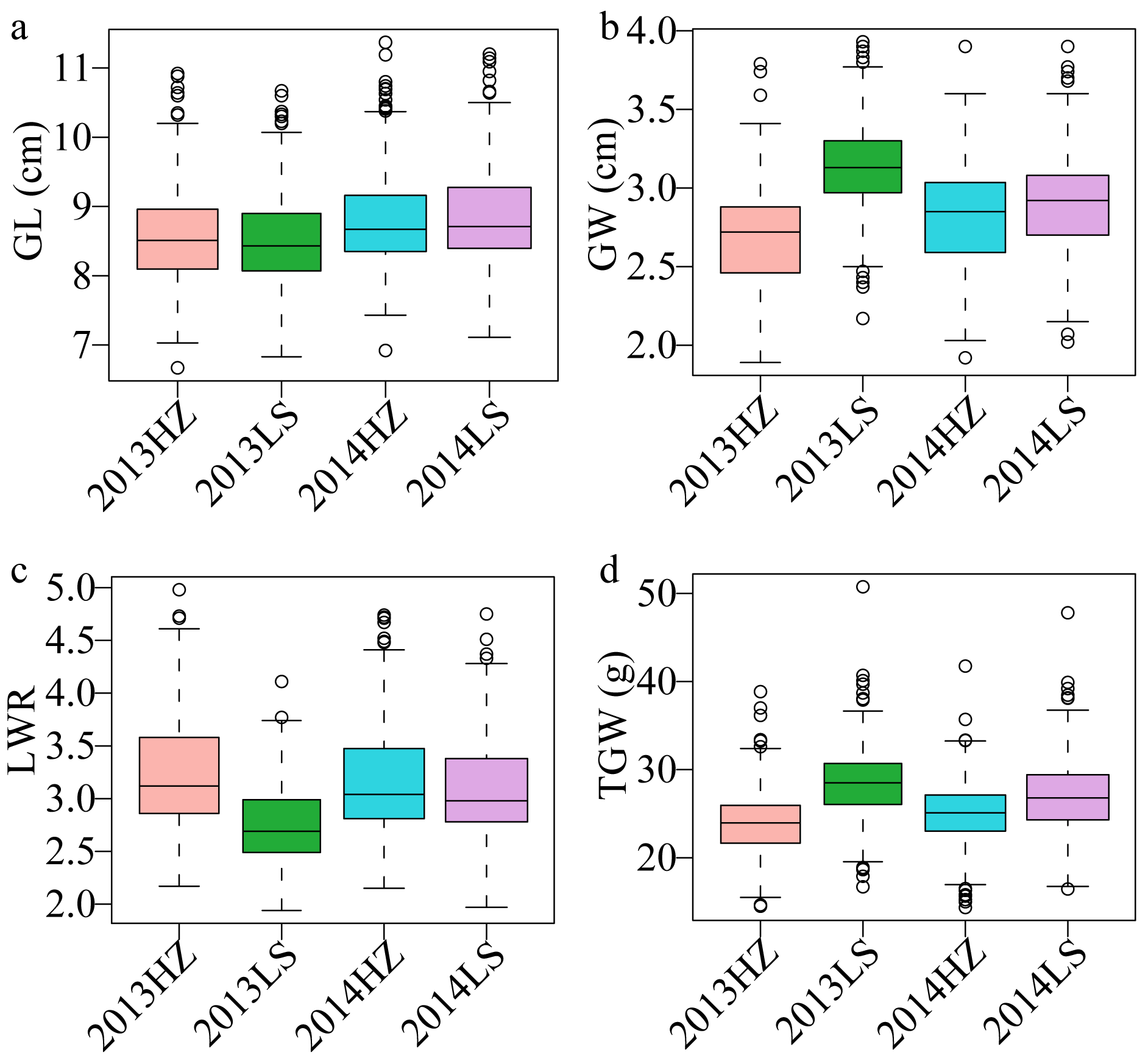

Supplement: Supplementary file 1 — Supplementary Figure S1: Box plot of four grain shape traits in four environments. (a) GL, grain length. (b) GW, grain width. (c) LWR, grain length–width ratio. (d) TGW, thousand grain weight. LS and HZ represent Lingshui and Hangzhou, respectively (TIFF 471 kb) [file 425_2016_2548_MOESM1_ESM.tif]
